# Monitoring change in volume of calcifications in juvenile idiopathic inflammatory myopathy: a pilot study using low dose computed tomography

**DOI:** 10.1186/s12969-016-0123-3

**Published:** 2016-11-29

**Authors:** Maria Ibarra, Cynthia Rigsby, Gabrielle A. Morgan, Christina L. Sammet, Chiang-Ching Huang, Dong Xu, Ira N. Targoff, Lauren M. Pachman

**Affiliations:** 1 Division of Pediatric Rheumatology, Children’s Mercy Hospital , 2401 Gillham Road, Kansas City, Missouri 64108-4619 USA; 2 Department of Medical Imaging, Ann & Robert H. Lurie Children’s Hospital of Chicago, Chicago, IL USA; 3 Department of Radiology, Northwestern University Feinberg School of Medicine, Chicago, IL USA; 4 Cure JM Center of Excellence, Stanley Manne Research Institute affiliated with Ann & Robert H. Lurie Children’s Hospital of Chicago, 225 East Chicago Avenue, Box 212, Chicago, IL 60611 USA; 5 Joseph J. Zilber School of Public Health, University of Wisconsin, Milwaukee, WI USA; 6 The Department of Internal Medicine, The University of Oklahoma, Norman, OK USA

**Keywords:** Computed Tomography (CT), Calcification volume, Juvenile idiopathic inflammatory myopathy, Overlap syndrome, Calcification

## Abstract

**Background:**

Dystrophic calcifications may occur in patients with J uvenile Idiopathic Inflammatory Myopathy (JIIM) as well as other connective tissue and metabolic diseases, but a reliable method of measuring the volume of these calcifications has not been established. The purpose of this study is to determine the feasibility of low dose, limited slice, Computed Tomography (CT) to measure objectively in-situ calcification volumes in patients with JIIM over time.

**Methods:**

Ten JIIM patients (eight JDM, two Overlap) with calcifications were prospectively recruited over a 2-year period to undergo two limited, low dose, four-slice CT scans. Calculation of the volume of calcifications used a CT post processing workstation. Additional patient data included: Disease Activity Scores (DAS), Childhood Myositis Assessment Scale (CMAS), myositis specific antibodies (MSA), and the TNFα-308 promoter region A/G polymorphism. Statistical analysis utilized the Pearson correlation coefficient, the paired *t*-test and descriptive statistics.

**Results:**

Ten JIIM, mean age 14.54 ± 4.54 years, had a duration of untreated disease of 8.68 ± 5.65 months  MSA status: U1RNP (1), PM-Scl (1), Ro (1, 4 indeterminate), p155/140 (2), MJ (3), Mi-2 indeterminate (1), negative (3). 4/8 JDM (50%) were TNF-α-308 A+. Overall, the calcification volumes tended to decrease from the first to the second CT study by 0.5 cm^3^ (from 2.79 ± 1.98 cm^3^ to 2.29 ± 2.25 cm^3^). The average effective radiation dose was 0.007 ± 0.002, 0.010 ± 0.005, and 0.245 mSv for the upper extremity, lower extremity and chest, respectively (compared to a standard chest x-ray-- 0.02mSV effective dosage).

**Conclusion:**

We conclude: 1) the limited low dose CT technique provides objective data about volume of the calcifications in JIIM; 2) measuring the volume of calcifications in an extremity is associated with minimal radiation exposure; 3) This method may be useful to evaluate the efficacy of therapies for JIIM dystrophic calcification.

## Background

In children with JIIM, such as Juvenile Dermatomyositis (JDM) and Overlap Syndrome, dystrophic calcifications are a common and debilitating complication. The reported calcifications in JDM range from 71% [1] to 8% [2] with 40% most frequently cited [3]. These calcifications usually occur in children with chronic inflammation and hypoxia associated with JIIM, which includes JDM, Polymyositis and Overlap Syndromes as well as in patients with other rheumatic diseases such as Scleroderma [4] and Systemic Lupus Erythematosus [5, 6].

Plain radiography is effective for the detection of calcinosis and the categorization of morphological patterns of calcification [7]. Although radiography is recommended for the initial imaging of calcinosis, it fails to evaluate objectively the volume of calcifications. Case reports have employed different types of whole body scans (scintigraphy using Technetium methylene diphosphonate (Tc-99 m MDP) and Tc-99 m pyrophosphate and Strontium nitrate) in an attempt to identify the location of the calcifications and to provide a quantifiable assessment of their extent, as well as to develop a method to monitor the child’s therapeutic response [8]. Scintigraphic evaluation using Tc-99 m MDP can effectively delineate sites of dystrophic calcifications in JDM and it is more sensitive in detecting visceral calcifications than plain radiographs [9]. However, scintigraphy has failed to provide a quantitative estimation of the volume of the calcification. In contrast, micro CT and synchrotron x-ray diffraction studies of calcified deposit samples from four children with the diagnosis of JDM characterized the microstructure of calcinosis, and demonstrated excellent sensitivity with respect to quantitation of amount and spatial distribution of minerals in these calcifications samples [10]. These studies suggested that CT could be used to measure the calcifications occurring in the soft tissues of children with JIIM, as this method had been effective in the experimental mouse model [11].

The purpose of this pilot study was to determine the feasibility of the use of low dose, limited slice CT as an objective measure of in-situ calcification volume, over time, in patients with JIIM.

## Methods

### Patient population

Approval was obtained from the Ann & Robert H. Lurie Children’s Hospital of Chicago Institutional Review Board to perform this prospective study (IRB#2008-13316). Inclusion criteria for study enrollment consisted of a diagnosis of JIIM and documentation of moderate to severe calcifications as determined by the assessor on clinical evaluation. One hundred and fifty JIIM patients were followed in our clinic during the time of the study. Of those, 14 (9.3%) had moderate to severe calcification; ten of these children were available for study; they gave their consent and were enrolled in the study. Eight of these children had severe calcifications and fulfilled the Peter and Bohan [12] criteria for definite/possible JDM and two of the patients had Overlap Syndrome and moderate calcifications. The duration of untreated disease was given by the patient's family. Each of the ten cases had two scans over a 2 year period. For consistency purposes, a single individual, the principal investigator (PI), marked the specific accessible area of the calcification to be scanned. The PI performed a complete physical examination; anatomic landmarks of the area were noted as a reference point for future scans.

### Laboratory assessment

#### TNF-α-308 promoter polymorphism

The A/G polymorphism in the −308 promoter region was determined by PCR, following established methodology [13].

#### Myositis Specific Antibody (MSA)

Each patient’s sera was analyzed at diagnosis and periodically thereafter, as new antibodies were recognized, by the Oklahoma Medical Research Foundation Clinical Immunology Laboratory, following methods previously outlined [14, 15].

### Clinical assessment

#### Disease Activity Scores (DAS)

The patient’s status with respect to 1) total DAS score; 2) skin involvement (DAS-S) as well as, 3) muscle strength and endurance (DAS-M) was assessed at the time of every visit according to a standard protocol [16].

#### The Childhood Myositis Assessment Score (CMAS)

An estimate of performance was obtained at every visit by a physical therapist, who was an independent observer [17].

### Imaging of calcifications

A limited four-slice CT was performed on a Siemens Somatom Sensation 64 CT scanner in the area of greatest calcium burden and utilized 100kVp, 100 mAs, 3 mm reconstructed slice thickness. Only the limb of interest was included in the scan field of view. For example, examination of the arm was performed in an image “arms-over-head” stance instead of alongside of the body to limit radiation exposure of the torso. The area of soft tissue calcifications was determined by thresholding the calcifications from the range of Hounsfield units of the calcifications (114–3017 Hounsfield units) using software on a Siemens CT workstation (MMWP, Siemens Medical Solutions, Malvern, PA). No sedation was required. There was a mean interval of 7.18 ± 2.33 months between the first scan and second scans. The computed tomography dose index (CTDI) and the dose length product (DLP) were reported by the scanner. The DLP was used to estimate the patient effective dose using published methods [18, 19] for the chest and lower extremities. Due to a paucity of data on the subject, estimates of the upper extremity effective dose were performed by a physicist based on published conversion factors for lower extremities [18, 19].

### Statistical analysis

Presentation of demographic and baseline characteristics used descriptive statistics. A paired t-test analyzed volume changes of calcifications in JIIM patients over time. Pearson correlation coefficient was used to determine the correlation between change in calcification volume and duration of active disease.

## Results

### Demographics and disease state

The demographics of the ten patients enrolled in this proof of principle study are presented in Table 1. The patients had active inflammatory disease for a mean of 8.68 ± 5.65 months. At the time of both scans, the patients still displayed symptoms of moderately active JIIM. Their DAS-S and DAS-M at the first scan were 6 ± 2.3 and 3 ± 3.7 respectively; (maximal DAS-M = 11, maximal DAS-S = 9; normal = 0 for both DAS-S and DAS-M) with a CMAS of 43 ± 7.4 (normal CMAS = 52). At the time of the second scan, their disease activity demonstrated very little improvement (DAS-S = 5 ± 2.2, DAS-M = 3 ± 2.7, CMAS = 44 ± 9.5). Two of the ten patients had Overlap Syndrome, one each positive for either Pm/SCL or U1RNP indeterminate. The remainder of the group was classified as JDM (4 Ro indeterminate; 1 Mi-2 indeterminate; three MJ positive; two p155/140 positive, one Ro positive; three negative for MSA). Of the ten subjects, eight had JDM and 4/8 (50%) were positive for TNF-α-308 GA polymorphism, the rest were GG, which was consistent with our previous findings [20]. Before entering the study, the patients had been given a variety of medications including; methotrexate (*n* = 7), hydroxychloroquine (*n* = 6), cyclosporine (*n* = 4), mycophenolate mofetil (MMF) (*n* = 4), prednisone (*n* = 6), intravenous methylprednisolone (*n* = 2) and intravenous gamma globulin (IVIG) (*n* = 2). During the study, the following medications were added: prednisone (*n* = 2), intravenous methylprednisolone (*n* = 3) and MMF (*n* = 3). All the children with calcifications were placed on at least two medications, but only five had been given a high dose intermittent pulse methylprednisolone. By end of the study, a total of six children had been given MMF, which did improve their rash [21]. Of note, the four patients for whom cyclosporine was proscribed stated that they were non-compliant with respect to the cyclosporine and had low serum levels of the drug.

**Table 1:** Clinical features of patients with juvenile idiopathic inflammatory myopathy

	Gender	Diagnosis	Duration of untreated disease (DUD) (mo)	Location of calcification	MSA status	TNF-α-308	Age at scan 1 (years)	Age at scan 2 (years)
Case 1	F	JDM	2.00	Arm	Ro indeterminate, MJ+	GG	11.25	11.71
Case 2	M	JDM	2.99	Forearm	Ro indeterminate, MJ+	GG	13.94	14.43
Case 3	F	JDM	2.00	Forearm	Ro indeterminate, p155/140+	GA	10.93	11.43
Case 4	M	JDM	0.36	Tibia	Mi-2 indeterminate	GA	16.32	16.86
Case 5	F	JDM	14.32	Knee	Negative	GG	8.89	9.48
Case 6	F	JDM	16.99	Arm	Negative	GG	20.58	21.17
Case 7	F	OVERLAP	0.00	Elbow	PMScl+	GG	13.72	14.35
Case 8	F	JDM	11.99	Knee	Negative	GA	20.06	20.73
Case 9	F	OVERLAP	10.05	Chest	U1RNP indeterminate, Ro indeterminate, MJ+	GG	20.27	20.93
Case 10	F	JDM	20.04	Arm	Ro+, p155/140+	GA	9.46	9.88

### Calcifications and CT scanning data

The calcifications were located as follows: three above the elbow, two in the forearm, one at the elbow, one in the area of the tibia, two around the knee and one in the chest wall. The average CTDI and DLP for the upper extremity (*n* = 12), lower extremity (*n* = 6) and chest (*n* = 1) are presented in Table 2.

**Table 2:** Average radiation dose estimates by CT scan location

	Total number of scans	Average CTDI mGy (stdv)	Average DLP mGy^a^cm (stdv)	Average effective dose mSv (stdv)
Upper extremity	12	4.322 (1.656)	16.133 (4.770)	0.007 (0.002)
Lower extremity	6	4.283 (0.581)	18.167 (3.488)	0.010 (0.005)
Chest^a^	1	4.200	17.000	0.245

^a^missing radiation information from first scan

**Fig. 1:**
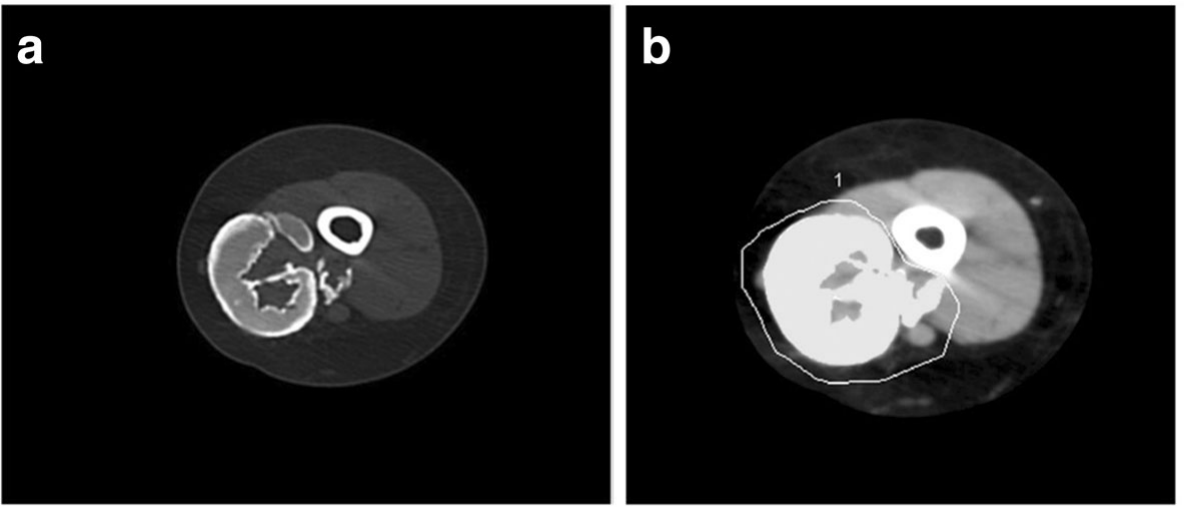
**a** Axial CT image of the upper arm of a child with JDM, showing a large cluster of calcifications medially. **b** The same axial CT image showing the calculated calcification volume highlighted in gray

Table 2. The CTDI and DLP are relatively similar for upper and lower extremities scanned in this study but the chest has a higher effective dose than the extremity studies. It is important to note, for comparison, that the comparable average effective radiation dose for a 15 year old child’s standard chest x-ray is 0.02 mSv [22], compared to the average effective doses above for the extremities of 0.007 and 0.010mSV), where the majority of the calcifications occur. Figure 1a presents an axial CT image of the upper arm showing a large cluster of calcifications medially, while Fig. 1b depicts the same axial CT image showing the calculated calcification volume highlighted in gray.

### The volume of the calcifications

**Fig. 2:**
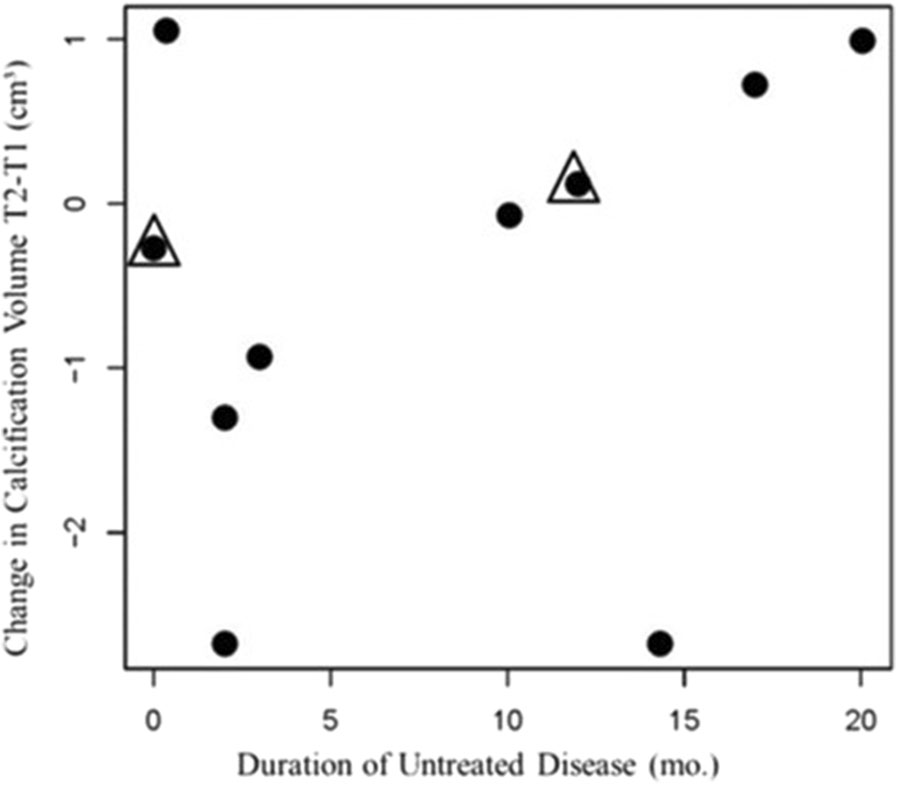
Black dots represent children with JDM. Black dots in a triangle represent two children with overlap syndromes (Pm-Scl and U1RNP)

**Fig. 3:**
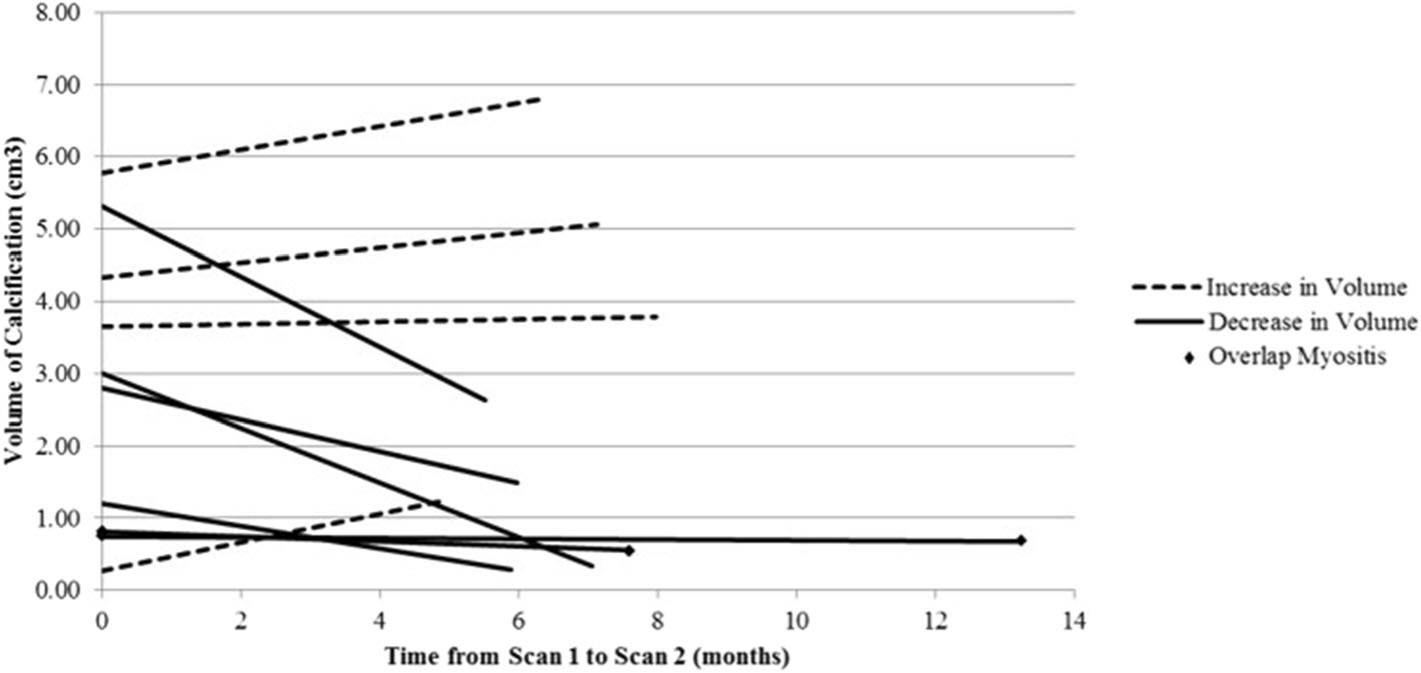
Change in the volume of calcifications: Six of the ten patients, who were compliant, did have a decreased calcification volume from 2.32 ± 1.77 cm^3^ on the first scan to 1.00 ± 0.92 cm^3^ on the second scan 6 months later scan with an average decrease of 1.32 cm^3^. The other four non-compliant patients had variable changes in the volume of their calcifications

There was a positive association of the change of the volume of calcification and DUD although this association was not statically significant (correlation 0.27, *p* = 0.46 Pearson correlation coefficient), (Fig. 2). The average size of calcifications at the first scan was 2.79 ± 1.98 and 2.29 ± 2.25 cm^3^ at the second scan, for an average decrease in size of 0.51 ± 1.38 cm^3^ (approximately 18%). However, the 6/10 who were compliant with their medications, did have a decrease in calcification volume from 2.32 ± 1.77 cm^3^ on the first scan to 1.00 ± 0.92 cm^3^ on the second scan, which was a measurable average decrease of 1.32 ± 1.14 cm^3^ (Fig. 3).

## Discussion

The present study represents the first pilot study to evaluate objectively the volume of calcifications in patients with JIIM over time. The mechanisms controlling the pathophysiology of calcifications remain poorly understood. Studies of the composition of these calcifications have shown that hydroxyapatite is the main mineral component, as well as calcium carbonate [23, 24]. Other bone matrix proteins such as osteonectin, osteopontin and bone sialoprotein have been documented [24], in addition to members of the integrin family [25]. Reported risk factors for the development of calcinosis include delayed diagnosis and treatment along with inadequate levels and duration of immunosuppressive therapy, [26] suggesting that prolonged inflammation contributes to tissue injury which promotes the calcium deposition. A common feature among all the children in this study was the persistent and chronic course of an active inflammatory process, which may be a major factor in the development/progression of calcifications in children with myositis [20]. Thirty percent of the JIIM group had a substitution of A at the TNFα-308 promoter region, which is similar to the 27–30% frequency in the general population, while 50% of the patients with JDM (4/8) were positive for the A substitution [20].

This study was not designed to test the efficacy of specific agents to elicit change in the volume of the calcifications, but to document that the single slice CT gives sufficient information to evaluate and compare outcomes. Our analysis of 20 CT scans of patients with JIIM and calcifications during a 2 year period while they were given immunosuppressive therapy showed a tendency for the specific calcification to decrease in volume in that time frame. In this group, 6/10 had positive MSAs, of whom 3/8 JDM were positive for one of the more frequently occurring MSAs, anti-MJ, reported to be associated with calcification [27].

We are not aware of any previous study that has used an objective method to measure the volume of calcifications embedded in the soft tissue of children with JIIM. For patients with systemic sclerosis, multidector computed tomography with multiplanar format has been employed successfully [28]. Experimentally, microcomputed tomography was employed to measure the volume of calcification in Abcc6 deficient mice [29] and to monitor the rate of resolution of induced calcification in mice with defective macrophage function [11].

Effective radiation dose in CT is dependent on three primary factors: the amount of radiation necessary to achieve the desired contrast between tissues, the extent of the body that is included in the field-of-view and the types of organs exposed to the radiation. The exceptionally low radiation dose needed to acquire the images in this study is a result of the favorable constitution, size, and location of JIIM calcifications. First, by their very nature –the deposits contain calcium (and are therefore comparatively radiopaque)– the calcifications are a strong contrast when compared to surrounding soft tissue (similar to bone). For this reason, very little radiation dose is needed to achieve excellent subject contrast and subsequent volume delineation; this was reflected in the reported CTDI value, which was low. Second, the physical size of the calcifications was easily captured in a 12 mm z-axis length, therefore limiting the radiation exposure to a very small anatomical region, which is reflected in the low DLP of this study. Finally, the JIIM calcifications in this study were located primarily in the extremities, which are relatively radio-insensitive. The radiosensitivity of cells increases with the reproductive rate and decreases with the level of differentiation, therefore the nerve and muscle tissue in the extremities is the least radiosensitive tissue in the body. The radiosensitivity of the exposed tissue is included in the calculation of effective dose in this report. Due to the low radiosensitivity of extremities to radiation, it would be optimal if future clinical studies were to select calcifications located in a limb as one inclusion criteria for evaluation of therapeutic efficacy using CT [30].

There are a few limitations to this pilot study: first, relatively few patients with calcifications were available at our Center for imaging. Another limitation is the lack of information to provide validation of this method (intra and inter rater reliability, validity, responsiveness). However, in a somewhat similar study, assessment of intrasubject change in lung tissue content over three CT scans was 2.75% +/−2.29% (mean and SD) [31]. In the present report, although their CT scans did not document a significant improvement in the volume of the calcifications in the 7 months between the first and second scan, the data do suggest that a longer interval between scans might be more helpful.

## Conclusion

We conclude that limited low dose CT provides a safe, objective measurement of calcifications in JIIM. Each study was found to deliver less radiation than a single chest xray due to the low dose technique, limited field of view, and relative radio-insensitivity of extremities [32]. We speculate that this method may be a useful research tool to monitor progression and regression of calcifications. The four slice CT method offers the possibility of detecting and documenting change in the volume of calcifications in children over time, and may be a useful adjunct in assessing the child’s response to therapy.

## Abbreviations

CMAS: Childhood myositis assessment scale
CT: Computed tomography
CTDI: Computed tomography dose index
DAS: Disease activity score
DLP: Dose length product
JDM: Juvenile dermatomyositis
JIIM: Juvenile idiopathic inflammatory myopathy
MSA: Myositis specific antibodies
